# B.M.A. and the National Scheme

**Published:** 1920-07-03

**Authors:** 


### B.M.A. and the National Scheme.

### Irish Proposal for State Medical Service.

The British Medical Association gave some consideration to the national scheme outlined in the interim report of the Medical Consultative Council of the Ministry of Health. The Association dealt mainly with general principles and submitted the whole question to the divisions to be considered later at a special representative meeting. Sir James Barr, with customary liveliness, declared that the Ministry of Health had no more to do with health than the man in the moon, and if we wanted A1 men we must breed them. It was therefore the business of the State to be engaged not with treatment, but with prevention of disease. His estimate of the cost of the scheme was a hundred millions a year for the first five years, and a hundred and fifty millions a year for the following ten years. It had been described as an ideal scheme, but an ideal scheme must be practicable. If you wanted to go to the moon, you had to turn it down, because it could not be done. Other delegates, however, contended that it was not only practicable, but a piece of statesmanlike construction which medical men ought to support, while the other side showed signs of fear lest the profession be jockeyed into an unsuccessful plan as they were jockeyed into the insurance plan. It seemed generally to be realised that, in any case, the scheme would take many years to materialise, and warnings were uttered against drifting away from individualism towards something in the nature of State action. It is too late in the day to declare against State action in a matter of such deep national concern as the public health, and what was probably meant by these warnings was objection to complete State action, which eliminated individualism. That is a point for which there is widespread support, and the Association indicates that view by reiterating its opposition to a whole-time State medical service. It is interesting in this connection to note that the majority report of the Irish Public Health Council, which is broadly on the lines of the English interim report, differs from this English plan by advocating a State medical service in Ireland. It is difficult to see why the cogent reasons against such a service should be any less applicable to Ireland than to England, and there is likely to be acute Irish controversy about it.

The B.M.A. approved of the establishment of primary health centres as the pivotal idea of the changes recommended, and passed a resolution declaring that in order to obtain an ideal system of medical and allied service, it was necessary that the prevention of disease as well as provision for its treatment should be based on domiciliary medical service, and that general practitioners should be actively encouraged in the practice of both.

There was considerable debate on the administrative machinery to carry out the recommendations, and it was resolved that the composition of the local health committee should provide for adequate representation of the medical profession residing in the area on the public bodies engaged in public health work, and that in each area there should be established a statutory medical committee.

